# Predicting Therapeutic Efficacy of Pharmacological Treatments in Children with Postural Orthostatic Tachycardia Syndrome: A Mini-Review

**DOI:** 10.3390/children10071093

**Published:** 2023-06-21

**Authors:** Siying Fan, Yaxi Cui, Ying Liao, Hongfang Jin

**Affiliations:** 1 Beijing Chao-Yang Hospital, Capital Medical University, Beijing 100020, China; fansiying2001@163.com; 2 Department of Pediatrics, Peking University First Hospital, Beijing 100034, China; tracycuiyaxi@gmail.com (Y.C.); jinhongfang@bjmu.edu.cn (H.J.); 3 State Key Laboratory of Vascular Homeostasis and Remodeling, Peking University, Beijing 100191, China

**Keywords:** postural orthostatic tachycardia syndrome, individualized treatment, midodrine, oral rehydration salt, metoprolol, children, predictor

## Abstract

Postural orthostatic tachycardia syndrome (POTS) is common in children, with an excessive increment in heart rate when moving from the supine to upright position. It has significant negative impacts on the daily life of pediatric patients. The pathogenesis of POTS includes peripheral vascular dysfunction, central hypovolemia, abnormal autonomic function, a high-adrenergic state, impaired skeletal-muscle pump function, the abnormal release of vasoactive factors, and autoimmune abnormalities. Therefore, the empirical use of pharmacological treatments has limited therapeutic efficacy due to the diversity of its mechanisms. A crucial aspect of managing POTS is the selection of appropriate treatment targeting the specific pathogenesis. This review summarizes the commonly used pharmacological interventions, with a focus on their predictive indicators for treatment response. Factors such as heart rate variability, plasma biomarkers, and cardiac-function parameters are discussed as potential predictors of therapeutic efficacy, enabling the implementation of individualized treatment to improve therapeutic effectiveness. This review consolidates the current knowledge on POTS, encompassing its clinical characteristics, epidemiological patterns, underlying pathogenic mechanisms, and predictive indicators for treatment response. Further research is warranted to enhance the understanding of POTS and facilitate the development of more effective therapeutic approaches for this challenging syndrome.

## 1. Introduction

Postural orthostatic tachycardia syndrome (POTS) is one of the common types of orthostatic intolerance (OI) [1]. Clinical symptoms of POTS include chronic OI and a heart rate (HR) increase upon transitioning from a supine to standing position [2]. According to a survey in the USA, almost one-third of children and adolescents under 18 years had symptoms of POTS [3]. Additionally, children with POTS often experience impairment in their physical and physiological health, which consequently has a detrimental impact on their overall life quality [4,5].

Besides receiving health education and non-pharmacological interventions, pediatric patients with POTS sometimes need pharmacological treatment [6,7]. The majority of clinicians rely on empirical treatment strategies to manage POTS, which have resulted in limited therapeutic efficacy in clinical practice [8,9]. This is likely due to the diversity of the underlying mechanisms of POTS in pediatric patients. POTS in children can result from several pathophysiological mechanisms, including peripheral vascular dysfunction, hypovolemia, autonomic dysfunction, a hyperadrenergic state, impaired skeletal-muscle pump activity, and autoimmune dysfunction [8,9,10]. Individualized treatments based on the different pathophysiological mechanisms underlying POTS holds greater promise than traditional empirical approaches [11]. This review indicates the latest advances in baseline hemodynamic parameters and biological indicators that can predict the clinical efficacy of POTS and provides an additional avenue for further exploration and inquiry into this syndrome.

## 2. The Development of POTS and Its Associated Manifestations

Symptoms related to POTS were initially observed by researchers in the 19th century, and the “postural orthostatic tachycardia syndrome” was introduced by Schondorf and Low to describe the condition. POTS is defined as a sustained increase in HR ≥ 30 beats per minute (bpm) after transitioning from the resting supine to the standing position or ≥120 bpm without a corresponding decrease in blood pressure (BP) within 3–10 min of standing and/or on a tilt table test [12,13]. However, the diagnostic criteria for HR increment of POTS in pediatric and adolescent patients differ from those of adult patients [14,15]. A cross-sectional study on the alteration of hemodynamic characteristics of the syndrome has suggested that the diagnosis of POTS should be made when the heart rate increases by ≥40 bpm during the position changes, associated with the presence of orthostatic symptoms. Likewise, a diagnosis of POTS can be established by observing a maximum heart rate of over 130 bpm, or 125 bpm for children aged 6–12 and 13–18 years old, respectively. This criterion has been incorporated into the clinical guidelines for the accurate identification of POTS in younger patients and has gained widespread clinical application [15].

The literature has identified three main POTS phenotypes based on different pathophysiologic mechanisms: (a) neuropathic POTS, (b) hyperadrenergic POTS, and (c) hypovolemic POTS [16]. Some experts have further categorized POTS into five subtypes, including the previously mentioned three forms and an additional two subtypes: joint hypermobility-related and immune-related [17,18]. Neuropathic POTS is characterized by reduced vessel vasoconstriction in the lower extremities. In neuropathic POTS, the dilation of blood vessels causes central blood to be redistributed while standing, leading to compensatory tachycardia in response to the orthostatic challenge [19]. The criterion of hyperadrenergic POTS is defined by an elevation in norepinephrine of greater than 600 pg/mL upon standing and a systolic blood pressure increase of greater than 10 mmHg following 10 min of standing [20]. Exactly opposite to neuropathic POTS, patients with hyperadrenergic POTS exhibit an increased activity of the sympathetic nervous system, and particularly an increased release of norepinephrine, resulting in increased peripheral vascular resistance [21]. One research on hyperadrenergic POTS revealed a reduction in the vasodilation of blood vessels with an excessive rise in plasma noradrenaline levels [21]. Patients with hypovolemic POTS typically exhibit reduced plasma volume, red blood cells (RBC), and urinary sodium excretion [22]. A study demonstrated that 28.9% of patients had a sodium excretion rate of less than 100 mEq/L within 24 h, which is indicative of low blood-volume status. Garland et al. found that a high-sodium diet increased plasma volume, lowered upright plasma norepinephrine level, and reduced the increment in heart rate during a standing test in patients suffering from POTS, which further supports the hypovolemic pathogenesis for POTS [23].

An epidemiological survey in the United States, involving 708 children with POTS, revealed a predominantly female population with a male-to-female ratio of 3.45:1. Moreover, symptoms often manifested in early adolescence [24]. Half of pediatric patients experienced syncope at a peak age of approximately 15 years [1]. Typical clinical manifestations of POTS include dizziness, headache, palpitations, chest tightness, pallor, and momentary blackouts associated with orthostatic posture [25]. Pre-syncope and syncope may occur in patients with severe POTS. In addition to posture-related symptoms, the patients may also present with various multi-systemic symptoms that are unrelated to body position, such as cognitive impairment, sleeping disorders, fatigue, attention deficits, and gastrointestinal dysmotility [26,27,28,29]. Chronic OI and accidental injury caused by recurrent syncope in children with POTS often restrict daily activities to varying degrees. POTS can considerably affect the physical and mental health of children.

## 3. Main Pathogenesis of POTS

The pathogenesis of POTS is closely associated with orthostatic physiology. When the body posture changes from a supine to upright position, the redistribution of blood occurs because of gravity. Increased blood flow is transferred to the circulation of the lower extremities and viscera, leading to a decreased return blood volume [30]. Under physiological conditions, hypotension due to the redistribution of blood flow can stimulate baroreceptors in the cardiovascular system [31], enabling the body to maintain a normal hemodynamic state through a series of compensatory mechanisms. Obstacles to any of these mechanisms can cause POTS. In such cases, there is a decrease in blood return and a subsequent decrease in cardiac output, leading to an inadequate blood perfusion to the brain. This insufficient cerebral perfusion manifests as clinical symptoms such as dizziness and syncope [32].

Pathophysiological studies have confirmed that POTS is a group of diseases mediated by multiple pathogenic mechanisms [10]. Currently, the pathogenesis of POTS mainly includes peripheral vascular dysfunction, central hypovolemia, abnormal autonomic function, high adrenergic state, impaired skeletal-muscle pump function, the abnormal release of vasoactive factors, and autoimmune-related mechanisms [13,14,18].

Peripheral vascular dysfunction is one possible cause of POTS [33]. Abnormal peripheral vasoconstriction or excessive dilation of the local vascular bed causes blood volume to pool in peripheral tissues, leading to distributive central hypovolemia when in the upright state [10]. This central hypovolemic state further reduces the return blood volume and cardiac output, leading to a compensatory posture-related acceleration of heart rate [20,22]. Subsequent investigations focused on the underlying pathogenesis of peripheral vascular dysfunction in pediatric POTS. The findings revealed an involvement of specific signaling molecules and vasoactive peptides in the development of abnormal vascular constriction and endothelial cell dysfunction. Significant differences were observed in the levels of endogenous nitric oxide (NO), hydrogen sulfide (H_2_S), and sulfur dioxide, as well as serum copeptin and C-type natriuretic peptide (CNP) between children diagnosed with POTS and controls [15].

Reduced blood volume was found in more than 70% of pediatric POTS, which could be exacerbated when upright [20,22]. Abnormal plasma concentrations of renin and aldosterone render the renin-angiotensin-aldosterone system (RAAS) incapable of compensating for hypovolemia [34]. Moreover, POTS patients with a high-sodium diet exhibited a lower plasma renin activity and aldosterone level than those with a low-sodium diet, accompanied with an increased blood volume and a decrease standing heart rate [23].

Moreover, POTS is associated with abnormal autonomic function. A plasma proteomic study revealed that the adrenergic activity was increased in the patients with POTS compared with healthy controls [35]. High plasma norepinephrine (NE) levels were found in some children or adults with POTS and were positively correlated with symptom severity [23,36]. Children with high circulating NE levels are in a “hyperadrenergic state”. Symptoms in children with POTS may also be accompanied by manifestations of hyperadrenergic stimulation such as pallor, tremors, palpitations, and hypertension. According to the genetic study of some cases, high plasma NE level is considered to be relevant to a dysfunctional norepinephrine transporter (NET) encoded by the SLC6A2 gene. Mutations of the SLC6A2 gene can result in a decrease in the NE reuptake between synapses and subsequent hyperadrenergic status. A histone deacetylase inhibitor GSK126 transcriptionally reactivated NET expression by abolishing trimethylation of lysine 27 of histone 3 on NET chromatin in leukocytes of patients with refractory POTS, which might further deepen the understanding of the epigenetic pathogenesis of hyperadrenergic POTS [37].

By contracting the lower-extremity muscles and activating their function as skeletal muscle pumps, the blood flow of the legs can be transmitted back to the heart. Investigators showed dramatically reduced leg blood flow in some pediatric patients with POTS [38]. Ischemia in the muscular tissue leads to a decrease in muscle mass or muscular atrophy, which affects the activity of the skeletal-muscular pump. A normal venous valve structure is necessary for skeletal-muscle pump function. Additionally, skeletal-muscular pump dysfunction may occur due to a venous valve defect or the congenital absence of a valve. The plasma myoglobin level in patients with POTS was lower than that in controls and the reduced myoglobin level in the patient was correlated with the severity of POTS, suggesting that there might be a relationship between abnormal skeletal muscle status and the development of POTS [39].

Recently, considerable attention has been paid to the autoimmune mechanisms underlying POTS as positive autoantibodies were found in some patients [40,41,42]. Li et al. mimicked the hemodynamic change in POTS patients using a rabbit model of adrenergic autoantibody-induced POTS, supporting an autoimmunity pathogenesis of POTS [43]. Furthermore, patients with POTS are more likely to develop autoimmune diseases such as Hashimoto thyroiditis, antiphospholipid syndrome, and rheumatoid arthritis [44]. Based on these findings, autoimmune dysfunction is considered one of the pathogenic mechanisms responsible for POTS. For example, Gunning et al. demonstrated that the antibodies against adrenergic and cholinergic G protein-coupled receptors could be detected in the sera of POTS patients [45]. Moreover, Kharraziha et al. found that the adrenergic α1 receptor could be activated by the serum from patients with POTS, which was correlated with the severity of OI in POTS patients [46].

## 4. Individualized Pharmacologic Therapies for Pediatric POTS: Predictors of Therapeutic Efficacy

Pediatric patients with POTS are often treated with non-pharmacological interventions, such as health education, exercises to improve autonomic function and sleeping therapy. The pharmacological treatment options include oral rehydration salts (ORS), midodrine hydrochloride, metoprolol, fludrocortisone and some other specialized therapies, such as droxidopa, desmopressin, erythropoietin and intravenous immunoglobulin (IVIG) [30,47,48,49,50]. Although there are medications available for the treatment of POTS, the empirical and unselected prescription has yielded poor clinical outcomes. Therefore, further enhancing the clinical therapeutic efficacy of POTS is currently a crucial clinical concern in the field. Thus, searching for stable, easy-to-use, and inexpensive biomarkers to predict the treatment response to different medications for patients with POTS has become a hot point in clinical research [11,51]. As such, further exploring clinical biomarkers for predicting the efficacy of the treatment of POTS and implementing individualized therapy have significant importance in improving the clinical outcomes of the patients.

### 4.1. Predictors for ORS Efficacy in Pediatric POTS

In children diagnosed with POTS, the redistribution of blood caused by gravity reduces venous return and cardiac output, resulting in changes in autonomic regulation and tachycardia. Medow et al. demonstrated that ORS treatment in pediatric POTS improved orthostatic tolerance and maintained cerebral blood-flow velocity [52]. However, only 20% of POTS patients responded to ORS when it was used unselectively. Actually, the effectiveness of volume expansion should be observed only in a subset of POTS patients with hypovolemia as the main pathogenesis [53]. Thus, the investigators successfully conducted a series of studies and discovered useful biomarkers predicting the therapeutic efficacy of ORS to implement individualized ORS therapy for children with POTS (Table 1).

#### 4.1.1. Level of 24 h Urinary Sodium Excretion

It has been confirmed that increasing blood volume through enhanced salt and fluid intake is beneficial for children and adolescents with POTS. However, only a subset of POTS patients are responsive to salt supplementation. Twenty-four-hour urinary sodium excretion was a reliable marker for assessing blood volume. Zhang et al. revealed that pediatric patients who responded to ORS exhibited low baseline twenty-four-hour urinary sodium excretion. Using urinary sodium excretion < 124 mmol/24 h before treatment as a cut-off value yielded a specificity of 93% and a sensitivity of 76.9% in predicting the effectiveness in the treatment of POTS [54]. In conclusion, 24 h urinary sodium excretion is a valuable biomarker and can prevent unnecessary salt supplementation, thereby avoiding the risk of excessive salt intake in affected individuals.

#### 4.1.2. Body Mass Index

Pediatric patients with POTS, characterized by low blood volume as the primary pathogenic mechanism, exhibit lower body mass index (BMI) compared with the patients diagnosed as vasovagal syncope and with healthy controls [58]. The administration of ORS can enhance blood volume and thereby alleviate symptoms. Identifying low-blood-volume patients through BMI measurements could potentially predict the efficacy of ORS. Li et al. explored the correlation between BMI and the therapeutic effect of ORS in pediatric POTS [55]. The findings suggested that the baseline BMI exhibited a strong predictive ability to predict the responsiveness to ORS treatment, yielding a high sensitivity (92%) and a specificity (82.8%). With ROC curve analysis, a BMI cutoff value of 18.02 kg/m^2^ was identified to predict the effectiveness of ORS. These results suggest that ORS treatment can effectively improve symptoms and yield favorable therapeutic outcomes in POTS patients with a lower BMI.

#### 4.1.3. Mean Corpuscular Hemoglobin Concentration

The alterations in RBC volume and count are implicated in POTS pathogenesis, potentially linked to hypovolemia. The hypovolemic state indicates low blood volume, including plasma volume (PV) and erythrocyte volume (EV). Hemocytometric indices can reflect the characteristics of erythrocytes, thereby reflecting the EV status. Lu et al. conducted a study to determine whether hemocytometric-related indices could predict the effectiveness of ORS in children with POTS. Baseline hemocytometric variables were measured before treatment, and then the patients were given a regimen in the form of a 3-month ORS treatment [56]. The results demonstrated that an increased mean corpuscular volume (MCV) as well as a decreased mean corpuscular hemoglobin concentration (MCHC) were independent risk factors for the development of POTS. Among children with POTS, those who responded positively to ORS treatment exhibited low baseline MCV and high MCHC compared to non-responders. The ROC study indicated an area under the curve (AUC) of 0.73 for the predictive value of MCHC. By using MCHC > 347.5 g/L as a threshold, the ability to predict the responsiveness to ORS treatment in POTS patients yielded an acceptable sensitivity and specificity of 68.8% and 63.2%, respectively. These findings suggest that MCV and MCHC measurements have potential as valuable indicators for predicting the response to ORS treatment in POTS patients.

#### 4.1.4. Baroreflex Sensitivity

Baroreflex sensitivity (BRS) is a quantitative index of evaluating the function of cardiovascular baroreceptor reflex, which plays a vital role in maintaining stable BP, HR, and blood volume within a physiological range, allowing the cardiovascular system to fit for different conditions [59]. Abnormalities in baroreflex function disrupt the normal regulatory mechanisms and contribute to the development of POTS [31]. In comparison to healthy children, Li et al. found that children with POTS exhibited significantly increased BRS values. Moreover, the baseline BRS acts as a valuable predictor of the prognosis of pediatric patients with POTS. A cutoff value of 17.01 ms/mmHg for BRS to predict the effectiveness of ORS plus conventional treatment in children with POTS yielded a sensitivity of 85.7%, a specificity of 87.5% and an AUC of 0.855. These findings highlighted the potential of BRS assessment as a convenient, cost-effective, noninvasive, and easily accessible tool for predicting the usefulness of ORS plus conventional treatment in individuals with POTS [57].

### 4.2. Predictors for Midodrine Efficacy in Pediatric POTS

The empirical use of midodrine for POTS is often not ideal because of the diverse pathogenesis and heterogeneity of the disease. Based on the pathogenesis of “peripheral vascular dysfunction,” the pediatricians identified several markers to predict the responsiveness to midodrine hydrochloride therapy and improved therapeutic outcomes (Table 2).

#### 4.2.1. Erythrocytic Hydrogen Sulfide Production

Recently, studies have confirmed the significance of NO, carbon monoxide (CO), and H_2_S as crucial endogenous gas signaling molecules. In the cardiovascular system, H_2_S is a potent vasodilator that relaxes the vascular smooth muscle. Erythrocytes are important source of endogenous H_2_S production. Since the cells are directly exposed to the internal environment, they are highly susceptible to changes in physiological conditions. Therefore, the quantification of endogenous H_2_S production can detect subtle and responsive changes in the body’s internal environment. This is made possible by the high-water solubility and lipophilicity of H_2_S, which allows for its free diffusion among cells and tissues [65].

Yang et al. measured the erythrocytic H_2_S production rates in pediatric patients with POTS and healthy controls. The plasma H_2_S level was increased in the patients in comparison with the controls. Moreover, responders to midodrine hydrochloride had a higher erythrocytic H_2_S production rate than the non-responders before treatment. The improvement in symptom scores, based on the clinical manifestations in the children before and after treatment, was positively correlated with baseline erythrocytic H_2_S production. According to ROC curve analysis, when erythrocytic H_2_S production was greater than 27.1 nmol/(min·10^8^ RBC), the sensitivity and specificity for predicting the satisfied therapeutic efficacy of midodrine reached 78.9% and 77.8%, respectively [61]. Furthermore, the value of baseline erythrocytic H_2_S production for predicting the long-term effect of midodrine hydrochloride in managing pediatric POTS was explored. The results showed that the group with higher erythrocytic H_2_S production [>27.1 nmol/(min·10^8^ RBC)] had a greater improvement in long-term symptom scores and higher symptom-free survival than the group with lower baseline erythrocyte H_2_S production. This study confirms the clinical value of erythrocytic H_2_S production in predicting the prognosis of children with POTS who received the treatment of midodrine hydrochloride. However, it is worth optimizing the drug dosage using further prospective clinical trials.

#### 4.2.2. Plasma Copeptin Level

Arginine vasopressin (AVP) release is increased due to excessive plasma osmotic pressure, reduced blood volume, or decreased systemic arterial pressure. Midodrine hydrochloride, as a selective α1-adrenoceptor agonist, can improve venous return by constricting peripheral blood vessels. Theoretically, plasma AVP level may be a potential predictor of therapeutic efficacy. However, AVP is extremely unstable in vivo, and its short biological half-life makes it difficult to accurately measure plasma AVP concentrations. Copeptin is a stable C-terminal fragment of AVP and correlates well with the production and release of AVP, indirectly representing the AVP concentration [66].

Compared to the healthy controls, pediatric patients with POTS had relatively high plasma copeptin levels. When patients were both treated with midodrine hydrochloride, patients who reacted well to the drug had higher copeptin levels than those who had no significant improvement of symptoms. Setting a 10.482 pmol/L or higher as the cutoff value of the baseline plasma copeptin level yielded a satisfactory predictive ability with a specificity of 76.5% and a sensitivity of 81.3% [61].

#### 4.2.3. Plasma Mid-Regional Pro-Adrenomedullin Level

Adrenomedullin (ADM) acts as a peripheral vasodilator. Animal experiments have suggested that ADM possesses robust vasodilatory properties and exerts anti-mitotic, natriuretic, and diuretic effects [67]. However, ADM has a short biological half-life and is mainly present in the form of binding proteins, which limits its clinical utility as a biomarker [68]. Mid-regional pro-adrenomedullin (MR-proADM) generates from ADM precursor in association with a production of ADM in a ratio of 1:1 by the enzymatic cleavage reaction. Compared with ADM, MR-proADM has a relative high stability. The characteristics as mentioned above allows the possibility of the application of plasma MR-proADM as an alternative index for evaluating the efficacy of individualized midodrine hydrochloride therapy, as it accurately reflects the concentrations of the product encoded by the ADM gene [68].

In a clinical study involving 57 pediatric POTS patients and 20 healthy children, an obvious difference in plasma MR-proADM levels was found between the two groups. The plasma MR-proADM level in the pediatric POTS responding to midodrine hydrochloride was increased compared with that of the un-responders (pre-treatment plasma MR-proADM levels: 66.0–91.0 pg/mL vs. 54.0–65.5 pg/mL, *p* < 0.01). With a baseline MR-proADM level > 61.5 pg/mL as the cutoff level, the predictive specificity and sensitivity reached 71.6% and 100%, respectively [62]. Through a 72-month follow-up, the predictive significance of baseline plasma MR-proADM levels for long-term symptom improvement in pediatric POTS receiving midodrine hydrochloride therapy.

#### 4.2.4. Flow-Mediated Vasodilation

A perfect endothelial function is critical for maintaining an appropriate vascular tone. Vascular endothelial cells modulate vascular tone by regulating the homeostasis between vasodilators and vasoconstrictors [69]. Endothelial dysfunction is mainly mediated by the abnormal NO generation and bioavailability and is involved in the pathophysiologic change in POTS [63]. Ultrasonography is an effective method for detecting flow-mediated dilation (FMD) to evaluate endothelial function. During detection, the basal end-diastolic diameter (Bd) and the blood flow of the brachial artery were measured and recorded. Subsequently, the artery was partially blocked for 5 min using a cuff. After relaxing the cuff, the filling velocity and maximum diastolic diameter (Fd) were measured during the FMD induced by NO production in the endothelial cells due to the shear stress generated by the transient high-speed state of blood flow. FMD can be expressed as flow-mediated arterial dilation: FMD = (Fd − Bd)/Bd × 100% [70].

Liao et al. investigated the differences in FMD between pediatric cases of POTS and controls. Firstly, the baseline FMD in children with POTS was significantly increased compared with controls. Subsequently, when both received oral midodrine hydrochloride therapy, the baseline FMD in the effective patients was increased compared with that of the ineffective ones. Finally, when an FMD > 9.85% was selected as a cutoff level, the specificity and sensitivity for the prediction for the treatment response to midodrine hydrochloride were 77.8% and 71.6%, respectively, after treatment with midodrine hydrochloride for 1 month [63]. The sensitivity and specificity increased slightly when the follow-up period was prolonged to 3 months. The measurement of FMD is non-invasive, easy to perform, and relatively acceptable for children and their families.

#### 4.2.5. Blood Pressure Changes in Standing Test

Standing test induces OI in children with POTS. During the test, the children were required to lie quietly in a supine position. During the test, the researcher recorded the basal heart rate and blood pressure (BP), including systolic blood pressure (SBP) and diastolic BP (DBP), while collecting a routine electrocardiogram (ECG). After 10 min, the children were instructed to stand, with heart rate, BP and ECG constantly being monitored. The appearance of a positive response or complement after 10 min of standing indicated the termination of the test [15]. Deng et al. performed a study to investigate the correlation between BP changes during the standing test and the efficacy of midodrine hydrochloride in 110 pediatric POTS patients. Prior to the initiation of oral midodrine hydrochloride therapy, variations in SBP and DBP were recorded following changes in body position. The children then received oral midodrine hydrochloride treatment at a dosage of 2.5 mg/day and the therapy lasted for 6 months. The results showed that the decrease in baseline SBP and DBP during the standing test before treatment in the responders was greater than that in the non-responders. Using the baseline decrease in SBP ≤ 0 mmHg before treatment as the value, the predictive sensitivity and specificity for the effectiveness of midodrine hydrochloride in patients with POTS were 60% and 80%, respectively. Using the baseline decrease in DBP ≤ 6.5 mmHg during the standing test as a cutoff point yielded the sensitivity and specificity of 63% and 92%, respectively, in the prediction of the effectiveness of midodrine hydrochloride [64]. Moreover, the baseline upright change in blood pressure in BP was capable of predicting long-term outcomes in pediatric POTS patients who received midodrine hydrochloride, as shown in the subsequent study.

#### 4.2.6. Subtypes of POTS

The noradrenergic activity in patients with neuropathic POTS and with hyperadrenergic POTS may significantly differ. An increasing number of studies focused on stratifying the participants based on their pathophysiologic phenotypes to better understand the treatment efficacy of midodrine hydrochloride on different groups of POTS patients [42]. Midodrine hydrochloride is expected to have a greater effect on improving the hemodynamic abnormalities in neuropathic POTS by promoting vasoconstriction, reducing venous capacitance, and thereby decreasing orthostatic tachycardia. However, its effects on hemodynamic improvement in hyperadrenergic POTS are limited due to the different pathophysiology compared with that of neuropathic POTS. Ross et al. revealed that midodrine hydrochloride was an effective drug in the treatment of neuropathic POTS but had limited efficacy for hyperadrenergic POTS [71]. After midodrine hydrochloride treatment, tachycardia was alleviated in neuropathic POTS patients. However, the elevated heart rate response in the hyperadrenergic POTS cases remained after midodrine hydrochloride treatment. In neuropathic POTS patients with vascular dysfunction, the use of midodrine hydrochloride might be likely effective.

### 4.3. Predictors for Metoprolol Efficacy in Pediatric POTS

Metoprolol is one of the most commonly prescribed β-adrenergic receptor blockers. For the POTS patients with an overactivated sympathetic activity as the primary pathophysiologic mechanism, metoprolol reduces heart-rate stimulation, counters the effects of elevated catecholamine levels, and alleviates symptoms by targeting β-adrenergic receptors. Therefore, the symptomatic improvement observed with the empirical usage of β-adrenergic receptor blockers is limited to unselected patients with POTS [72]. The scientists investigated if there were any useful baseline markers to forecast the effectiveness of metoprolol in cases of POTS before treatment. Below, we have summarized the literature on clinical investigation about the individualized treatment of metoprolol in pediatric POTS (Table 3).

#### 4.3.1. Orthostatic Plasma Norepinephrine Level

Increased sympathetic activity is a main mechanism underlying POTS. The elevation of upright plasma norepinephrine levels may induce impaired vasoconstriction via the baroreceptor reflex [76]. Zhang et al. found that increased upright plasma norepinephrine levels were associated with severe symptoms in pediatric and adolescent POTS patients. Also, there existed a correlation between standing plasma norepinephrine levels and the efficacy of metoprolol in children with POTS [36]. The results demonstrated that using a cutoff value of 3.59 pg/mL for plasma norepinephrine levels had the sensitivity and specificity of 76.9% and 91.7% in the prediction of the efficacy of metoprolol treatment for POTS.

#### 4.3.2. Plasma CNP Level

CNP is a polypeptide that belongs to the natriuretic peptide family. The physiological effects of CNP are widely reported, including decreasing blood pressure, relaxing the blood vessel, inhibiting the proliferation of smooth muscle cells, promoting natriuresis and diuresis, and suppressing RAAS [77]. CNP possesses several advantages, including easy detection, stable circulating levels, and convenient storage. Studies have shown that CNP levels in the plasma of POTS patients contribute to vasodilation, increased heart rate, and elevated concentrations of catecholamines in the circulatory system. Lin et al. found a significant positive correlation between the severity of POTS, including symptom scores, the increase in HR from supine to upright position and the maximum standing heart rate, and plasma CNP levels in children with POTS. A baseline plasma CNP concentration greater than 32.55 pg/mL was found to be a strong predictor of metoprolol efficacy in children with POTS with a predictive sensitivity of 95.8% and a specificity of 70% [73].

#### 4.3.3. Heart Rate Variability

Heart rate variability (HRV) could reflect the regulation of the autonomic nervous system and the balance between sympathetic and parasympathetic nervous activity. Studies have shown that HRV is useful for reflecting changes in autonomic nervous system tone [78]. A meta-analysis of the 717 POTS patients and 641 healthy controls showed that the time domain measures of HRV in POTS patients were lower than those in controls [79]. Inbaraj et al. conducted a cohort of POTS patients in India and demonstrated that the resting low frequency/high frequency ratio in POTS patients was higher than that in healthy controls, further supporting the theory that the imbalance between parasympathetic and sympathetic activity was involved in the pathophysiological mechanisms for POTS [80]. Wang et al. found that baseline HRV was a valuable predictor of metoprolol efficacy in pediatric POTS. A serial-parallel analysis of HRV indices revealed that baseline triangular (TR) index ≤ 33.7 and standard deviation of NN intervals (SDNN) index ≤ 79.0 ms served as critical measures, with a sensitivity of 85.3%, a specificity of 81.8%, and an accuracy of 84.4%, respectively [74].

#### 4.3.4. HR and HR Difference

Wang et al. explored the role of HR and HR differences during standing test in predicting the symptom improvement in pediatric POTS treated with metoprolol [75]. The results showed HR indices, including HR 5 min, HR 10 min, ΔHR 5 min, and ΔHR 10 min could predict the efficacy of metoprolol in POTS. In detail, for HR 5 min, a threshold of ≥110 bpm yielded a sensitivity of 82.50% and a specificity of 69.23% in predicting the satisfied efficacy of metoprolol in POTS. Regarding HR 10 min, a threshold of ≥112 bpm has a similar predictive ability. While selecting a threshold of ΔHR 5 min ≥ 34 bpm, they obtained a relatively highly predictive specificity of 89.47%. As for ΔHR 10 min, a threshold of ≥37 bpm exhibited a high sensitivity of 97.56% in the prediction of the effect of metoprolol in POTS cases.

### 4.4. Treatment with Ivabradine in Pediatric POTS

An extensive literature research was made and just a few studies specifically examining the biomarkers to predict the efficacy of ivabradine in the treatment of adult with POTS were found [81,82]. Further studies could be performed in order to assess efficacy and biomarkers target of ivabradine in children with POTS should be performed [30,47,83].

## 5. Conclusions and Perspective

In summary, recent studies have made significant progress on baseline markers in predicting the efficacy of pharmacological treatment options for managing pediatric POTS, including hemodynamic parameters and bioactive factors. Before treatment is initiated, measuring the baseline values of these markers is reflective of the possible main mechanisms underlying POTS. This can help pediatricians identify children with POTS who are more sensitive to different pharmacological therapies, which is useful for implementing individualized treatment. Further trials are needed to confirm the clinical predictive value of markers that have been shown to have significant advantages in terms of a high predictive sensitivity or specificity, a low cost, and convenience of detection. Finally, subsequent large-sample multicenter prospective cohort and randomized controlled studies are needed to obtain more robust evidence to evaluate the efficacy, determine the treatment dose, and assess the adverse drug reactions to further optimize individualized treatment with midodrine hydrochloride for POTS in children.

## Figures and Tables

**Table 1:** Biomarkers predicting the treatment effectiveness of ORS in pediatric postural orthostatic tachycardia syndrome.

Biomarkers	Cut-Off Value	Predictive Sensitivity (%)	Predictive Specificity (%)	References
24 h urinary sodium (mmol/24 h)	<124	76.9	93	Zhang et al. [54]
Body mass index (kg/m^2^)	<18.02	92	82.8	Li et al. [55]
Mean corpuscular hemoglobin concentration (g/L)	>347.5	68.8	63.2	Lu et al. [56]
Baroreflex sensitivity (ms/mmHg)	>17.01	85.7	87.5	Li et al. [57]

**Table 2:** Biomarkers predicting the effectiveness of midodrine hydrochloride in pediatric postural orthostatic tachycardia syndrome.

Biomarkers	Cut-Off Value	Predictive Sensitivity (%)	Predictive Specificity (%)	References
Erythrocytic H_2_S production [nmol/(min·10^8^ RBC)]	>27.1	78.9	77.8	Yang et al. [60]
Plasma copeptin (pmol/L)	>10.482	81.3	76.5	Zhao et al. [61]
Plasma MR-proADM (pg/L)	>61.5	100	71.6	Zhang et al. [62]
Flow-mediated dilation (%)	>9.85	71.6	77.8	Liao et al. [63]
ΔBP (mmHg)	ΔSBP < 0	60	80	Deng et al. [64]
	ΔDBP < 6.5	63	92	

RBC, red blood cell; MR-proADM, mid-regional pro-adrenomedullin; Δ, change; BP, blood pressure; SBP, systolic blood pressure; DBP, diastolic blood pressure.

**Table 3:** Biomarkers predicting the treatment effectiveness of metoprolol in pediatric postural orthostatic tachycardia syndrome.

Biomarkers	Cut-Off Value	Predictive Sensitivity (%)	Predictive Specificity (%)	References
Orthostatic plasma norepinephrine (pg/mL)	>3.59	76.9	91.7	Zhang et al. [36]
Plasma C-type natriuretic peptide (pg/L)	>32.55	95.8	70	Lin et al. [73]
Heart rate variability	TR index < 33.7 and SDNN index < 79.0 ms	85.3	81.8	Wang et al. [74]
HR 5 min (bpm)	>110	82.5	69.23	Wang et al. [75]
HR 10 min (bpm)	>112	84.62	69.70	
ΔHR 5 min (bpm)	>34	85.29	89.47	
ΔHR 10 min (bpm)	>37	97.56	64.86	

TR, triangular; SDNN, standard deviation of NN intervals; HR, Heart rate; Δ, Change.

## Data Availability

Not applicable.
